# T176. REDUCED WHITE MATTER ‘CONNECTIVITY’ IN THE SPLENIUM OF THE CORPUS CALLOSUM IN TREATMENT-RESISTANT SCHIZOPHRENIA

**DOI:** 10.1093/schbul/sby016.452

**Published:** 2018-04-01

**Authors:** Idaiane Assunção-Leme, André Zugman, Luciana Monteiro de Moura, João Ricardo Sato, Deyvis Rocha, Bruno Bertolucci Ortiz, Cristiano Noto, Vanessa Ota, Sintia Belangero, Rodrigo Bressan, Andrea Parolin Jackowski, Ary Gadelha

**Affiliations:** 1 Laboratório Interdisciplinar de Neurociências Clínicas(LiNC); 2 Laboratório Interdisciplinar de Neurociências Clínicas(LiNC), Universidade Federal de São Paulo(UNIFESP); 3 Universidade Federal do ABC (UFABC); 4 Universidade Federal do ABC, Laboratório Interdisciplinar de Neurociências Clínicas(LiNC), Universidade Federal de São Paulo(UNIFESP); 5 Universidade Federal de São Paulo (UNIFESP)

## Abstract

**Background:**

Resistance to treatment affects up to 30% of patients with schizophrenia (SCZ). Current criteria for treatment-resistant schizophrenia (TRS) require failure to respond to two antipsychotic trials for adequate dose and duration. Clozapine is the only antipsychotic that is more effective to treatment resistant patients. Increasing evidence suggest that TRS may represent a subgroup of patients with distinct biological signature. Brain dysconnectivity was proposed as a major feature of schizophrenia and more intense in TRS patients. Earlier identification of TRS may anticipate the clozapine trial and, thus, reduce disability and treatment costs. In our study, we investigated whether there were differences in white matter integrity among first episode of psychosis (FEP), treatment-resistant schizophrenia (TRS), and non treatment-resistant schizophrenia (NTRS) patients.

**Methods:**

Diffusion-tensor brain MRI images were obtained for 34 TRS (19 males), 50 NTRS (26 males) and 35 FEP individuals (18 males), on a Siemens 1.5T MRI scanner. Treatment resistance was defined as persistence of moderate to severe symptoms, after failure to respond to 4–6 week trials of at least two different antipsychotic medications in adequate doses (equivalent to at least 400 mg/day of chlorpromazine or 5 mg/day of risperidone). All participants were receiving antipsychotic medication. All TRS patients were in clozapine use. Analysis of diffusion parameters was performed using a tract-based spatial statistics (TBSS), yielding a total two contrasts: i) mean FA is lower (or higher) in the TRS compared to the FEP, ii) mean FA is lower (or higher) in the NTRS compared to the FEP corrected for multiple comparisons using family-wise error (FWE) < 0.05. Gender and age were used as covariates.

**Results:**

FEP patients were younger than TRS (mean±SD; 27.2 ± 7.93 y/o vs 37.06 y ±7.98 y/o;t=5.08, p <0.001) and NTRS (27.2 ± 7.93 y/o vs 37.71 y ±11.18 y/o; t=4.57, p<0.001) patients. Reduced in FA value was observed in the splenium of the corpus callosum (CC) in TRS patients when compared to FEP (47,598 voxels and thresholded at p<0.05). No differences between NTRS and FEP patients were observed.

**Discussion:**

Our results showed reduced FA value in the splenium of the CC in TRS when compared to FEP. The splenium of corpus callosum connects the temporal and occipital cortices, and have been previously associated with schizophrenia, but not specifically to treatment resistance in schizophrenia. Our data might suggest that patients with resistance to treatment have inefficiency in the connectivity of the white matter between these regions. Further studies will be required to replicate these findings and to explore the significance of white matter changes in the brain in order to determine if these are consequence of disease progression or related to clozapine exposure.

